# A Review of Data for Compound Drought and Heatwave Stress Impacts on Crops: Current Progress, Knowledge Gaps, and Future Pathways

**DOI:** 10.3390/plants14142158

**Published:** 2025-07-13

**Authors:** Ying Li, Ketema Zeleke, Bin Wang, De-Li Liu

**Affiliations:** 1 CMA·Henan Agrometeorological Support and Applied Technique Key Laboratory, Zhengzhou 450003, China; 2 Henan Institute of Meteorological Sciences, Zhengzhou 450003, China; 3 Zhengzhou Agrometeorological Field Observation and Research Station of Henan Province, Zhengzhou 450003, China; 4 Zhengzhou Agrometeorological Field Science Experiment Base of CMA, Zhengzhou 450003, China; 5 Gulbali Institute for Agriculture, Water and Environment, Charles Sturt University, Wagga Wagga, NSW 2650, Australia; kzeleke@csu.edu.au (K.Z.); bin.a.wang@dpi.nsw.gov.au (B.W.); de.li.liu@dpi.nsw.gov.au (D.-L.L.); 6 School of Agricultural, Environmental and Veterinary Sciences, Charles Sturt University, Wagga Wagga, NSW 2650, Australia; 7 New South Wales Department of Primary Industries, Wagga Wagga Agriculture Institute, Wagga Wagga, NSW 2650, Australia; 8 Hawkesbury Institute for the Environment, Western Sydney University, Richmond, NSW 2753, Australia

**Keywords:** compound extreme events, drought and heat stress, lab experimental data, field trial data, Earth system data, statistical data, simulated data

## Abstract

Compound drought and heatwave (CDHW) events have shown a marked increase under global warming, posing significant challenges to crop productivity. This review systematically categorizes key input and output datasets utilized across diverse research frameworks that investigate the impacts of CDHW stress on crops. The data are organized across multiple spatial scales—from site-specific and field-level measurements to regional and global assessments—and span various temporal dimensions, including historical records, present conditions, and future projections. These datasets include laboratory experiments, field trials, Earth system observations, statistical records, and model simulations. By employing a structured and integrative approach, this review aims to facilitate efficient data access and utilization for researchers. Ultimately, it supports improved data integration, cross-study comparability, and cross-scale synthesis, thereby advancing the assessment of climate change impacts on agricultural systems.

## 1. Introduction

Compound or concurrent extreme events (CEs) refer to the occurrence of two or more extreme events either simultaneously or in sequence [1]. CEs result in devastating impacts at a scale far greater than what any one of these extremes could have caused in isolation [2]. Among the various types of CEs, those associated with droughts and heatwaves are the primary focus of this review. Compound drought and heatwave (CDHW) events have significantly impacted various aspects of agricultural production [3], such as crop yields, vegetation productivity, water scarcity, and animal health.

Although climate extremes have always been a common cause of crop failures worldwide [4], droughts and heatwaves are particularly destructive events affecting crop production [5,6]. Moreover, future climate change, driven by anthropogenic global warming, is expected to intensify extreme weather events and alter the risk of CEs [7], potentially increasing the frequency of crop failures [8]. While some high-latitude regions may become more conducive for crop growth, many major crops in agricultural areas will likely experience negative effects [9].

With human-induced climate forcing, there has been a significant increase in CDHW events globally [10], recently doubling the likelihood of experiencing both warm and dry years at the same location, compared to the 1961–1990 baseline, and increasing the probability of simultaneous extreme heat and drought in key agricultural and pasture regions [11]. Regional analyses reflect similar patterns. For instance, the frequency of CDHW events has significantly increased across much of the United States between 1960 and 2010, accompanied by a statistically significant shift in their distribution [12]. Similarly, from 1951 to 2020, many regions in the Mediterranean basin experienced a significant increase in dry and warm months, especially in the summer and spring [13]. In Central Asia, the frequency of CDHW events rose over time from 1981 to 2018, with an expansion of their affected area, especially in the eastern and southwestern regions [14]. In addition to the observed increases in the frequency, severity, and duration of CDHW events at global and regional scales in recent decades, projections indicate that these events will continue to rise across most global land areas throughout the 21st century [15].

CDHW extremes can cause compounding damage to crop yields, with the plant defense response under this combination exhibiting new patterns, rather than the simple additive effect seen when experiencing drought or heat stress alone [16]. The increased likelihood of CDHW conditions could lead to particularly severe and unique impacts on crops [17,18]. In nine of the top ten maize-producing countries, CDHW events have the highest likelihood of causing maize yield reduction [19]. Additionally, the combined heat and drought stress index can account for 42% of the global wheat yield variability, and this correlation holds true at the national level as well [20]. Moreover, the risk to the global population and cropland from CDHW events is projected to increase under the SSP2-4.5 and SSP5-8.5 scenarios across different future periods, particularly in North America, Europe, Africa, and Oceania [21].

The response of plants to a combination of distinct abiotic stresses is unique and cannot be inferred directly from their individual responses to each stress applied separately [22]. In most studies, the CDHW stress exhibits synergistic effects, where the combined stress is more severe than either individual stress or their additive effects, causing significantly greater adverse impacts on crop growth and productivity [23,24,25,26,27]. However, some studies have observed antagonistic effects for certain traits, where the combined stress is less severe than either individual stress, their additive impact, or hypo-additive effects, where the combined stress is more significant than the individual stresses but still less than their total combined impact [24,28]. In certain cases, when one stress dominates, the combination of stresses does not further increase the damage to plants [29]. Therefore, understanding the impacts of CDHW stress on crops necessitates comprehensive and specialized data that cannot be derived from single-stress experiments, despite the wealth of experimental data available for individual stress factors. Moreover, experiments addressing compound events are inherently more complex than those focusing on single stresses. While previous studies have produced valuable experimental data on CDHW stress, these findings have yet to be systematically summarized in prior reviews.

Building on previous studies, this review not only includes experimental data on the impacts of CDHW stress on crops but also summarizes the input data involved, depending on the different research frameworks. These research frameworks include the following: (1) Tailoring crop responses to specific CDHW situations [22]: Conducting lab experiments or field trials to uncover the mechanisms of plant responses to CDHW stress, assessing crop resilience, and guiding adaptations such as breeding, variety selection, and planting date adjustments. (2) Historical trends analysis and crop failure attribution: Analyzing long time-series data or typical cases to explore the spatial and temporal characteristics of CDHW events on a large scale, aiming to explain crop failure due to these events [30] or attribute the yield consequences to historical trends [31]. (3) Future projections and crop risk assessments: Emphasizing large-scale risk assessments of crop failures caused by CDHW events, including assessments of past and current conditions [32], and valuating future crop risks via climate projections [33].

Building on the outlined research framework, this review categorizes data related to the impacts of CDHW stress on crops into several types. These include site-scale laboratory experimental data and field-scale trial data (framework 1), Earth system datasets and statistical records (framework 2), and simulated outputs (framework 3). This review synthesizes available data on CDHW stress impacts on crops across various spatial scales—ranging from site and field to regional and global—and temporal ranges, encompassing historical, present, and future periods.

**Figure 1:**
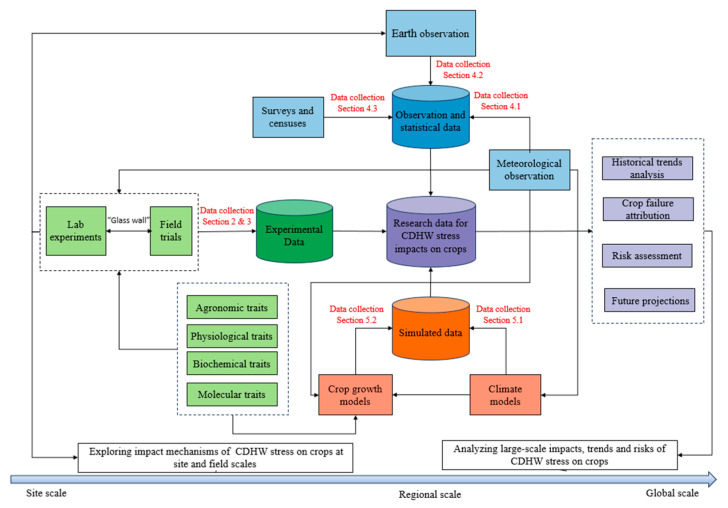
A workflow diagram representing the outcome of the review from the collected data.

The review strategy, which aims to provide a systematic perspective to help researchers effectively access and utilize existing datasets, is represented as a workflow diagram in Figure 1. In the diagram, purple elements represent the research data relevant to CDHW stress impacts on crops and the corresponding research aims. Green elements indicate the experimental data discussed in this review, including lab experimental data, introduced in Section 2, and field trial data, described in Section 3. Blue elements denote the Earth system data and statistical data, discussed in Section 4. Orange elements represent the simulated data, introduced in Section 5, with specific datasets referenced in the corresponding subsections as indicated in the figure. By emphasizing spatial and temporal interconnections, our review strategy promotes data integration, enhances the comparability of research findings, and enables cross-scale data synthesis, ultimately advancing more efficient and impactful research in this field.

## 2. Lab Experimental Data

### 2.1. Experimental Data Under Control

In this review, we refer to data obtained from conducting CDHW stress treatments in growth chambers and greenhouses as “lab experimental data.” This definition encompasses both situations where crops are continuously grown under controlled conditions within growth chambers and greenhouses throughout their entire growth stages, and those where crops initially grow under field conditions before being exposed to compound stress under controlled conditions.

In the first situation, controlled facilities eliminate short- and medium-term variability in weather and soil conditions throughout the entire crop growth process, allowing for clear interpretation and reproducibility of the data [34]. Growth chambers provide more precise control over abiotic stress conditions compared to greenhouses; therefore, most stress treatments are conducted in growth chambers [35]. In some experiments, growth chambers are used across all stages of crop growth [29,36], while in others, plants are initially grown in greenhouses before being transferred to growth chambers for abiotic stress application [37]. However, in some cases, stress treatments are also conducted in greenhouses [38,39]. A comparative study on heat treatments in glasshouses and growth chambers indicates that, despite the superior control in growth chambers, variations in diurnal temperature curves, relative humidity, or radiation during heat stress had a minimal impact on the study’s outcomes [24].

In the second situation, emphasis is placed on growing plants under field conditions both before and after the application of stress in controlled facilities. These studies also focus on replicating local meteorological conditions during stress treatments conducted in greenhouses and growth chambers [40]. In one case, prior to stress application, crop pots were maintained outdoors with netting. Stress was then applied in growth cabinets for a duration of three days to assess tolerance effects, after which the pots were returned to their original randomized outdoor positions [41]. In another case, potted plants were initially grown outdoors under a canopy. Stress conditions were then simulated in an artificial growth chamber based on the climatic conditions of the study location. After the stress treatments, the pots were returned to natural conditions until the plants matured [42].

### 2.2. Crop Species, Varieties, and Stress Treatments

The data acquired from studies on CDHW stress conducted in the lab involve a variety of crop types. Wheat (*Triticum aestivum* L.) is the most extensively studied crop [38], followed by tomato (*Solanum lycopersicum* L.) [43]. For other crops, experimental results are available for crops such as barley (*Hordeum vulgare* L.) [44], maize (*Zea mays* L.) [45], oilseed rape (*Brassica napus* L.) [46], chili (*Capsicum annuum* L.) [47], rice (*Oryza sativa* L.) [26], potato (*Solanum tuberosum* L.) [48], lucerne (*Medicago sativa* L.) [36], soybean (*Glycine max* (L.) Merr.) [49], tobacco (*Nicotiana tabacum* L.) [50], eggplant (*Solanum melongena* L.) [29], and citrus (*Citrus* spp.) [37]. Additionally, Arabidopsis thaliana has been used as a model plant in several studies. The extent of research cases is influenced not only by the agricultural importance of different crops but also by the feasibility of conducting experiments under laboratory conditions. While crops like wheat and tomato have comparatively more experimental data from lab studies, others, such as maize, have relatively limited data from lab experiments under CDHW stress.

In the selection of tested varieties, some studies use a single variety, typically a widely cultivated local variety [51] or a high-yield cultivar [42]. Most studies, however, involve comparisons among different cultivars, wild types, genotypes, and transgenic lines [52]. These comparisons primarily focus on genotypes with contrasting stress susceptibilities, including comparisons between drought-tolerant cultivars and high-temperature-tolerant cultivars [53], and between stress-tolerant genotypes and stress-sensitive genotypes [54,55].

The timing for initiating stress treatments in crops typically follows two shared patterns. One way is based on days after sowing (DAS) or days after transplanting [54,56]. The other way focuses on specific growth stages of the crops [40,44,51]. In some experiments, the timing of drought stress occurred earlier than that of heat stress [24], and the exposure durations of the two stresses were inconsistent [50,57].

Most lab-based experiments set a single-stress treatment period. These periods range from high-intensity stress treatments conducted over a few hours [43,57] to short- to medium-term stress lasting several days or weeks [41,58], as well as stress applied until physiological maturity [38,51]. In some cases, stress durations are also determined by cumulative stress thresholds; for example, the stress treatment is terminated when the stress temperature time (STT) exceeds a specific value [24]. Multiple data points are collected during and after acclimation [48,58,59], and in some cases, additional data is collected following the recovery period [56].

CDHW conditions in lab settings typically involve four main treatments: control, heat stress, drought stress, and CDHW stress [23,38]. This design provides valuable data for comparing the impacts of single and combined stress events on plant performance. However, some studies have only applied CDHW stress [50]. Some lab experiments have also introduced supplementary variables to help deepen the understanding of CDHW stress and its interactions with other factors affecting crops. The interaction between elevated CO_2_ and CDHW stress has been investigated in the lab for wheat [60], tomato [43], and oilseed rape [61]. Some experiments have expanded the understanding of interactions between different nutrient levels and CDHW stress in crop systems [42,46], while other studies have investigated the effects of CDHW stress under acid [49], salinity [59], and melatonin treatments [40]. Experimental data have also been collected on plant performance under biological amendments during CDHW stress, such as the impact of inoculating arbuscular mycorrhizal (AM) fungi [57].

### 2.3. Data Collection of Traits in Lab Experiments

In lab experiments focusing on CDHW stress, the measured traits generally fall into the following categories: phenotypic traits, growth and physiological traits, biochemical traits, yield traits, and gene expression. Most studies generate data covering a variety of physiological and biochemical traits, such as leaf water content, leaf pigment content, stomatal conductance, enzymatic activities, photosynthetic rate, malondialdehyde content, soluble sugars, and proline accumulation [35,36,49]. Some studies place additional emphasis on phenotypic measurements, such as plant height, leaf area index (LAI), and growth rate [54], while others focus on grain yield and quality analysis, assessing traits such as grain size, weight, and nutrient content [62]. Additionally, some experiments focus on gene expression analysis, providing data on the regulation of stress-responsive genes and pathways [44]. These experimental results capture the responses of different crops to CDHW stress across various biological levels, providing valuable insights into the regulatory mechanisms underlying stress impacts and adaptation.

It is important to note that experimental data from different tested varieties show significant variability [48]. This highlights the diverse responses of different genotypes and cultivars under stress conditions, underscoring the importance of selecting suitable varieties in stress research. Given the significant variation in responses among varieties under stress, it is essential to thoroughly understand the characteristics of the selected cultivars and genotypes when referencing and using existing experimental data for a specific crop type.

## 3. Field Trial Data

### 3.1. Trial Data Under Management

In this review, the term “field trial data” refers to data obtained from applying CDHW stress under field conditions with specific management practices. Here, field conditions do not necessarily imply plants grown outdoors in soil without any physical barriers towards the environment; the one-plant-one-pot approach is still included. The emphasis lies in the application of management measures in natural outdoor conditions, contrasting with the controlled conditions provided in the lab.

There is significant variation between the two in terms of control over biotic and abiotic factors. Field trial data typically provide a clear description of the geographical locations of the experimental sites. These trials examine CDHW stress conditions and control and single-stress conditions. CDHW conditions are based on the climatic and weather patterns at the experimental sites, with management practices applied to one or both of the variables, water and heat, during the trials.

Water supply is typically managed through a combination of keeping off precipitation and controlling irrigation. Keeping off precipitation can be achieved by planting during the dry season [28] or using rainout shelters at sites with natural rainfall [63]. Rainout shelters block precipitation, but the rainy season also brings high humidity and low light conditions, which can affect crop evapotranspiration. Therefore, field trials conducted during the dry season tend to provide data that more closely resemble natural drought stress conditions. Irrigation management is implemented through regular irrigation during non-stress periods, with the reduction or cessation of water supply during drought stress periods [64]. For crops like rice, this also involves draining water from the plots [65].

Heat settings are typically managed in three main ways. One approach involves selecting different plant growth sites. Each location–period combination is considered as a separate environment [66]. Another method involves manipulating the planting dates at different times at the same site, typically including both normal and late planting treatments [67]. The third method involves using heating devices. The main approaches include installing infrared radiators suspended above the plots subjected to heat stress [68] and covering high steel structures with polyethylene film to create a greenhouse effect [69]. Additionally, in one trial, the heating system utilized a mobile greenhouse in the field [63]. Among the three methods used to induce heat stress, the first method introduces uncertainty in the results due to variations in soil type and other climatic conditions across different sites. The second method is influenced by short-term weather differences between treatments. The third method, which uses heating devices, can impact light exposure and the field microclimate.

### 3.2. Crop Species, Varieties, and Stress Conditions

Field trial data on CDHW stress have also been accumulated for a wide range of crop species, including wheat [70], peanut (*Arachis hypogaea* L.) [28], maize [71], lentil (*Lens culinaris* Medik.) [67], soybean [72], chickpea (*Cicer arietinum* L.) [25], sorghum (*Sorghum bicolor* (L.) Moench) [73], and rice [65]. The types of crops in field trials are somewhat fewer than those in lab experiments, with maize being the most studied crop in field trials. Some field trials have chosen a single variety, such as the most popular maize hybrids in China [68]. Most of the trials selected different genotypes to provide comparative data among varieties, primarily including drought-tolerant, drought-sensitive, heat-tolerant, and heat-sensitive genotypes [74]. Field trials may also screen a larger range of varieties, such as a peanut trial with 268 genotypes [28] and a wheat trial that selected a Recombinant Inbred Line population consisting of 167 lines [64].

In contrast to lab data, which is typically derived from different treatments conducted concurrently at a single-site experiment, field trial data is usually collected over multiple growing seasons, spanning two or more years and one or more sites. This can even include large-scale, multi-site joint experimental data. For example, one study on maize under CDHW stress used joint experimental data from 15 multi-environment trials conducted at field stations in Mexico, Kenya, Thailand, Zimbabwe, and India between 2008 and 2011 [75]. Field trial data has the characteristic of such large-scale, multi-site applications, which is rare in lab data.

In field trial data, in addition to including treatments for CDHW alongside control groups, some studies also provide contrasting data on either single drought stress [76], single heat stress [77], or both conditions for comparison [78]. However, the incidence of field trials providing a comparison between a single stress and CDHW stress is lower compared to lab experiments. Moreover, it is less common for field trials to incorporate additional variables beyond drought and heat stress. One example is a study that used a tunnel house to elevate CO_2_ levels, applying two different CO_2_ concentrations, four temperature treatments, and specific watering regimes on two wheat genotypes [70].

The timing for initiating stress treatments in the field also follows two approaches: based either on DAS [71] or the growth stages of the crops [79]. The latter approach is more commonly used, and for the same crop, different trials tend to focus on different growth stages. For example, in current field maize trials, CDHW stress is applied at various growth stages, such as the booting [64], tasseling [80], anthesis [78], or filling [68]. Furthermore, within a single trial, different time points for stress treatments may also be compared [81]. For example, in a trial on hybrid maize, heat stress, drought stress, and combined drought-heat stress were applied at the 3rd leaf stage, 12th leaf stage, and tasseling stage. The results showed that the yield reduction caused by high temperature, drought, and their combined stresses was most significant at the tasseling stage [63]. Additionally, treatment duration in field trials is typically longer than in laboratory experiments, ranging from stress applied over several days [68] to stress extending until physiological maturity [66].

### 3.3. Data Collection of Traits in Field Trials

Field trial data primarily focus on growth, physiological, and yield traits. Growth traits typically include plant height, leaf area index (LAI), biomass accumulation, and overall growth rate, which assess plant development under CDHW conditions. Physiological traits involve metrics such as leaf water content, stomatal conductance, transpiration rate, and photosynthetic efficiency, helping to evaluate how plants cope with CDHW stress under field conditions. Yield traits, a key aspect of field trial data, encompass grain size, number, weight, harvest index, seed filling rate, and nutrient content, which are crucial for assessing crop productivity, nutritional quality, and resilience to CDHW stress. Some field trial data also provide insights into biochemical traits, such as leaf pigment content, proline accumulation, antioxidant enzyme activity, and reactive oxygen species levels, though these are less frequently reported than in lab experiments. Moreover, gene expression data, which offer a deeper understanding of the molecular mechanisms behind CDHW stress tolerance, are relatively scarce in field trials.

## 4. Earth System Data and Statistical Data

### 4.1. Meteorological Data

The most crucial supporting data for studying CDHW events at regional and global scales are meteorological data corresponding to the relevant spatial areas, with precipitation and temperature being the key elements. Other important elements include evapotranspiration (ET), potential evapotranspiration (PET), solar radiation (SR), heat flux (HF), relative humidity (RH), specific humidity (SH), wind speed (WS), wind direction (WD), and soil moisture (SM). Based on these elements, hot and dry conditions can be characterized by defining metrics, such as maximum temperature, accumulated precipitation, and root zone soil moisture [82], or by calculating specific indices, including heatwave indices such as the standardized temperature index (STI) and the heat wave magnitude index (HWMI), as well as drought indices such as the self-calibrating Palmer drought severity index (SC-PDSI), the standardized precipitation index (SPI), and the standardized precipitation evapotranspiration index (SPEI) [83,84,85,86]. Meteorological datasets available for regional, national, and global CDHW events primarily include observation-based datasets, reanalysis datasets, and national weather services. The uncertainties and performance of different meteorological datasets can be found in some comparative studies [87]. The most commonly used datasets in the first two categories are shown in Table 1. Researchers often utilize one or more of these datasets. When multiple datasets are used, the goal is typically to validate the quality of a primary dataset [88] or to gather different elements from various databases [89].

Observation-based daily or monthly precipitation and temperature data are most widely used in large-scale compound event studies. Among these, the dataset sourced from the Climate Research Unit (CRU) at the University of East Anglia is the most frequently utilized. This dataset is generated based on data from over 4000 meteorological stations worldwide, and the monthly precipitation and temperature data, with a spatial resolution of 0.5° × 0.5°, are extensively utilized by researchers for studies at both global and national scales [8,90]. Additionally, PET data from this dataset [17], and the monthly self-calibrating Palmer drought severity index (scPDSI) series [83], are also commonly applied in the study of CDHW events. In some cases, when focusing on specific study areas, the data were downscaled from CRU to better suit local conditions [91]. Observation-based data from the Climate Prediction Center (CPC), with a spatial resolution of up to 0.25°, provide global daily data that is another commonly used source in compound event studies, with daily maximum and minimum 2 m air temperature data being used most frequently [92], while precipitation data [93] and climate indices are also retrieved from the CPC. In addition to the two widely used global comprehensive datasets, regional datasets like the European Climate Assessment & Dataset (EOBS) [30], and temperature-focused data from Berkeley Earth [18] and precipitation-focused data from the Global Precipitation Climatology Center (GPCC) [94], are also commonly used.

In addition, several regional multi-national datasets are available, including the APHRODITE project, providing daily precipitation and temperature data for East Asia (http://aphrodite.st.hirosaki-u.ac.jp/, accessed on 15 June 2025), the SA-OBS high-quality daily gridded meteorological dataset for Southeast Asia ( https://sacad.bmkg.go.id/, accessed on 15 June 2025), the CHIRPS precipitation dataset for global tropical and subtropical regions (https://www.chc.ucsb.edu/data/chirps, accessed on 15 June 2025), and the meteorological observation datasets for the Tibetan Plateau and mid-latitude regions provided by the TPDC (https://data.tpdc.ac.cn, accessed on 15 June 2025). However, research utilizing these datasets in the context of CDHW events remains limited, and further exploration is recommended.

Reanalysis data combines observational data from multiple sources with advanced models, providing richer meteorological variables and higher temporal resolution for CDHW event research. The ERA-5 reanalysis dataset (Copernicus Climate Change Service), provided by the European Centre for Medium-Range Weather Forecasts (ECMWF), is among the most widely used in compound event studies. It is primarily applied for hourly and daily temperature and precipitation data [95,96], with a spatial resolution of 0.25° × 0.25°. Additionally, variables such as SM, PET, SR, SH, and HF have also been frequently utilized from this dataset [97,98]. The Modern-Era Retrospective Analysis for Research and Applications, Version 2 (MERRA-2), provided by the National Aeronautics and Space Administration (NASA), and the JRA-55 climate dataset, provided by the Japan Meteorological Agency (JMA), are also commonly used reanalysis datasets in compound event studies [99,100,101]. There are new and successor datasets, such as JRA-3Q (https://jra.kishou.go.jp/JRA-3Q/index_en.html, accessed on 15 June 2025) [102], which have yet to be utilized in studies of CDHW events. Researchers are encouraged to stay informed about these updated data sources and to take the initiative in applying such novel datasets to CDHW research.

Datasets from national weather services are also a common source. Such studies are usually conducted at the national and sub-national level, utilizing station-level or gridded historical weather data provided by the country’s national meteorological agency. Various meteorological elements, primarily including precipitation and temperature, are sourced from datasets of multiple national meteorological services. Common sources include the China Meteorological Administration (CMA) (http://www.cma.gov.cn, accessed on 10 February 2025) for the China Meteorological Forcing Dataset [103] and the Daily Meteorological Dataset of Basic Meteorological Elements of China National Surface Weather Station [104]. In the United States, the National Oceanic and Atmospheric Administration (NOAA) (https://www.noaa.gov, accessed on 10 February 2025) provides several datasets, for example, the Climate Divisional Database [7], the Global Historical Climate Network (GHCN) [11], and Variable Infiltration Capacity simulations for the Continental United States (CONUS) [12]. National weather service data from other countries can also be accessed through the official websites of their respective meteorological agencies [97,105,106,107,108].

**Table 1:** The most widely used observation-based and reanalysis datasets in CDHW crop impact research.

Category	Dataset	Data Source	Variables	Temporal Coverage	Spatial Coverage	Temporal Resolution	Spatial Resolution	URL Accessed on 10 February 2025	Ref.
Observation-based	CRU TS	University of East Anglia	Comprehensive	1901–Present	Global	Monthly	0.5° × 0.5°	http://www.cru.uea.ac.uk/data	[109,110]
	CPC	CPC	Comprehensive	1981 to present	Global	Daily	0.25° × 0.25°	https://www.esrl.noaa.gov/psd	[111]
	E-OBS	C3S	Comprehensive	1950–Present	Europe	Daily/Monthly	0.25° × 0.25°	https://surfobs.climate.copernicus.eu/dataaccess/access_eobs.php	[112]
	Berkeley Earth	Berkeley Earth	Specialized- temperature	1750–Present	Global	Daily	1° × 1°	http://berkeleyearth.org/data	[113]
	GPCC	GPCC	Specialized- precipitation	1901–Present	Global	Daily	1° × 1°	http://gpcc.dwd.de/	[114]
Reanalysis	ERA-5	ECMWF	Comprehensive	1950–Present	Global	Hourly/Daily/Monthly	0.25° × 0.25°	https://cds.climate.copernicus.eu	[115]
	MERRA-2	NASA	Comprehensive	1980–Present	Global	Hourly/Daily/Monthly	0.5° × 0.625°	https://gmao.gsfc.nasa.gov/research/merra/	[116]
	JRA-55	JMA	Comprehensive	1958–2012	Global	6-hourly/Daily/Monthly	TL319	https://data.diasjp.net/dl/storages/filelist/dataset:204/lang:en	[117]

### 4.2. Remote Sensing Data and Products

In addition to meteorological data, Earth observation (EO) technologies, which acquire wide-area information on the Earth’s surface via remote sensing satellites, also serve as an important source of Earth system data for studying the agricultural impacts of CDHW events. Remote sensing techniques, such as multispectral, thermal, and microwave imaging, provide essential insights into changes in surface vegetation, hydrology, and temperature across multiple spatial scales, including field, regional, national, and global levels, even in regions where ground-based measurements are unavailable. Recent advancements in EO satellite missions, such as Landsat, Sentinel, and Soil Moisture Active Passive (SMAP), alongside processing platforms like Google Earth Engine (GEE), have significantly enhanced these capabilities, offering high-resolution spatial and temporal data. This wealth of data has been effectively applied to examine the spatiotemporal dynamics of CDHW events and their impacts on crops, focusing on the following categories:

Vegetation growth-related parameters: Vegetation indices, primarily the Normalized Difference Vegetation Index (NDVI) and Enhanced Vegetation Index (EVI), are used as proxies for assessing vegetation health, sourced from sensors, such as the Moderate Resolution Imaging Spectroradiometer (MODIS) (NASA, Greenbelt, MD, USA) [99,118] and the Advanced Very High-Resolution Radiometer (AVHRR) (NOAA, Silver Spring, MD, USA) [119]. LAI data from the MODIS MOD15A product [120], Global Inventory Monitoring and Modeling Studies (GIMMS), Global Land Surface Satellite (GLASS), and Global Mapping (GLOBMAP) datasets [90], as well as Gross Primary Production (GPP) from several remote sensing-based global datasets [17,101], are also commonly used to assess vegetation health and productivity.

Photosynthetic activity-related parameters: This category primarily involves solar-induced chlorophyll fluorescence (SIF), sourced from datasets such as TROPOMI SIF [121] and GOME-2A SIF [122], which are strongly correlated with plant photosynthesis rates and show potential for monitoring vegetation’s physiological responses to CDHW conditions. This category also includes datasets for photosynthetically active radiation (PAR) and the fraction of photosynthetically active radiation (fPAR) [123], which are essential for understanding photosynthetic processes in plants under CDHW stress.

Soil moisture-related parameters: This category primarily includes satellite-based surface and root zone soil moisture data from the Global Land Evaporation Amsterdam Model (GLEAM) [98,124], European Space Agency (ESA) Climate Change Initiative (CCI), NASA SMAP [125], the NNsm soil moisture raster dataset [126], and terrestrial water storage (TWS) data from the joint US–German (NASA-GFZ) Gravity Recovery and Climate Experiment (GRACE) satellite mission [123].

Evapotranspiration-related parameters: This category primarily includes daily actual evaporation, PET, and ET data obtained from remote sensing, sourced from GLEAM [88,127], the Simplified Surface Energy Balance Algorithm for Land (SSEB) [125], and the MODIS product MOD16A [128].

Satellite-Based LST, precipitation, and land cover data: This category includes Land Surface Temperature (LST) and precipitation data typically obtained through direct measurements from satellite sensors [129]. Commonly used datasets include the MODIS daily LST product MOD11A [100] and precipitation data from the Global Precipitation Measurement (GPM) mission [101]. It also includes land cover datasets, obtained from satellite imagery classification, such as the land cover data from the MODIS product MCD12Q [90].

In addition, commonly used datasets also come from various environmental data projects that integrate remote sensing data, ground-based observations, and meteorological model outputs. For example, soil moisture data from the Global Land Data Assimilation System (GLDAS) (https://disc.gsfc.nasa.gov/datasets/GLDAS_CLSM025_D_2.0/summary?keywords=GLDAS, accessed on 10 February 2025) has been widely used [18,130]. Other widely used datasets include the Global Available Water Content (AWC) dataset provided by NASA (https://webmap.ornl.gov/ogc/dataset.jsp?ds_id=548, accessed on 10 February 2025) [30], the global GPP dataset generated by the FLUXCOM project (https://www.fluxcom.org/, accessed on 10 February 2025) [131], the global surface runoff G-RUN ensemble dataset (https://doi.org/10.6084/m9.figshare.12794075.v1, accessed on 10 February 2025) [132], and the Global Standardized Precipitation Evapotranspiration Index (SPEI) dataset (https://spei.csic.es/spei_database/, accessed on 10 February 2025) [14].

### 4.3. Statistical Data

The statistical data discussed here focuses on the impacts of CDHW events on agriculture, primarily encompassing information derived from agricultural surveys and censuses related to cropping patterns, cultivated areas, and yields. These data are typically released regularly by governmental agencies and relevant organizations at various levels [133].

Firstly, statistical data relevant to CDHW studies can be obtained from official statistical, agricultural, and water resource departments within the countries of the study region. These agencies often publish national datasets through government portals. For example, such datasets are provided by agencies, including the United States Department of Agriculture (USDA) (http://www.nass.usda.gov/Quick_Stats, accessed on 15 June 2025) [124,134], the Directorate of Economics and Statistics, Ministry of Agriculture and Farmers Welfare, India [135], the Spanish Ministry of Agriculture, Fisheries, and Food (URL: https://www.mapa.gob.es/, accessed on 15 June 2025) [136], and the National Bureau of Statistics of China (URL: http://data.stats.gov.cn, accessed on 15 June 2025), among others [118].

Additionally, international organizations such as the Food and Agriculture Organization (FAO) (URL: http://faostat.fao.org, accessed on 15 June 2025) and the World Bank (URL: https://data.worldbank.org/, accessed on 15 June 2025) provide standardized cross-national statistical datasets, which serve as important data resources for large-scale regional analyses. Common sources include the annual country-level crop production data and irrigation data from the FAO [32,137] and the annual Eurostat crop yield statistics from the European Commission [138].

Some specialized agriculture databases have also been used, including a global gridded dataset at a 0.5° resolution, MIRCA2000 [139], which provides the growing seasons and monthly irrigated and rainfed crop area around the year 2000. Based on MIRCA2000, the Inter-Sectoral Impact Model Intercomparison Project Phase 2a (ISIMIP2a) has provided Dynamic MIRCA. There are several other well-known global datasets that have been utilized, such as the Harvested Area and Yields of 175 Crops Dataset (http://www.earthstat.org/harvested-area-yield-175-crops/, accessed on 10 February 2025) [140], the Crop Calendar Dataset (https://nelson.wisc.edu/sage/data-and-models/crop-calendar-dataset/index.php, accessed on 10 February 2025) [141], and the global dataset of historical yields for major crops (GDHY) [142], among others. Additionally, data sourced from integrated projects that compile multiple datasets, such as the Agricultural Model Intercomparison and Improvement Project (AgMIP) (https://www.agmip.org/, accessed on 10 February 2025), are employed [133].

Socioeconomic data related to agriculture has been obtained from statistical agencies of different political units [128]. Some specialized organizations, such as the NASA Socioeconomic Data and Applications Center (SEDAC) (https://sedac.ciesin.columbia.edu, accessed on 10 February 2025), provide global datasets covering a wide range of topics, including population, cropland, land use, urbanization, poverty, and the impacts of climate change [143]. Geospatial databases related to agriculture are also utilized, including the Emergency Events Database [5], the Natural Earth dataset (https://www.naturalearthdata.com/, accessed on 10 February 2025) [14], and the Harmonized World Soil Database (www.isimip.org/protocol, accessed on 10 February 2025) [144]. Additionally, data on certain compound dry and hot events, such as the economic loss from agricultural damage caused by the 2003 European summer heatwaves and drought, are sourced from relevant reports like the COPA-COGECA 2003 report (https://copa-cogeca.eu, accessed on 10 February 2025) [145].

## 5. Simulated Data

### 5.1. Climate Model Simulation Data

Climate model simulation data have been widely used to extend the temporal and spatial scope of research, allowing for highly controlled and repeatable comparative experiments under standardized conditions by varying scenario parameters. These data can be used to analyze the spatiotemporal evolution characteristics of CDHW events under different climate scenarios, providing fundamental support for attribution analysis of such impacts on crops. In particular, when combined with crop models or field observations, such data greatly enhance the understanding of the underlying pathways through which CDHW events affect agricultural production and improve predictions of future risks.

In existing studies on CDHW events, climate simulations are commonly conducted under the framework of the Coupled Model Intercomparison Project (CMIP), primarily using data from Phase 5 (CMIP5) and Phase 6 (CMIP6), accessed on 10 February 2025) [106,146,147,148]. Some studies have utilized meteorological data generated by a single Global Climate Model (GCM) [27,149], while ensemble simulations involving multiple GCMs are more widely adopted to address uncertainties in modeling and scenario projections [150,151]. Previous studies have identified specific GCMs that perform better in certain regions. These regionally well-performing models can be preferentially used in CDHW events research to improve the reliability of regional-scale analyses [152]. For example, one study assessed CDHW extremes over the contiguous United States, providing critical insights into the appropriate selection and interpretation of GCMs for future regional assessments [153].

Future climate projections are based on different greenhouse gas emission scenarios. Under the CMIP5 framework, many climate change studies have utilized the RCP4.5 and RCP8.5 scenarios [27], while some have applied a single RCP or other comparative scenarios [11]. In recent years, an increasing number of studies on compound hot and dry events have widely utilized data and experimental designs from CMIP6-endorsed MIPs [154], commonly incorporating multiple Shared Socioeconomic Pathways (SSPs), particularly SSP1-2.6, SSP2-4.5, SSP3-7.0, and SSP5-8.5 [155,156,157,158]. Among these, SSP3-7.0 has been receiving increasing attention due to its distinctive aerosol emissions [159].

Among the existing studies that utilize climate model simulation data for CDHW research, one of the notable applications is attribution analysis of historical events and the assessment of their potential recurrence under present and future climate conditions. For instance, one study used CMIP6 ensembles and HadGEM3-A data to analyze the 1976 CDHW event in the UK and found that the annual probability of similar compound events, historically around 1%, could increase to approximately 5% by the 2040s [149]. Another study employed temperature and precipitation outputs from the CESM Large Ensemble and other GCMs to investigate the agricultural impacts of 1930s-level droughts in the United States. Climate model simulations were used to construct warming scenarios and quantify the relationship between extreme climate conditions and crop yield reductions [150]. In Northwestern Europe, the summer of 1976 was characterized by an exceptional heatwave and drought. A study using observational records, CMIP5 coupled climate model simulations, and HadGEM3-A atmosphere-only simulations has shown that the joint probability of such extremely hot and dry summers has significantly increased since the 1970s [160].

Another major application of climate model simulation data is to assess the spatiotemporal distribution, occurrence probability, and changing trends of CDHW events. One study analyzed the global land-based CDHW events from 1951 to 2010 by comparing CMIP6 climate model outputs with multi-source observational data. It found that simulations with natural forcing alone significantly deviated from observations, highlighting the critical role of anthropogenic forcing in the observed increase in CDHW events [161]. Another study evaluated the changes in CDHW characteristics, such as severity, frequency, and spatial extent, over both historical and future periods in CMIP6 simulations for the Indian subcontinent [106]. A separate investigation used multi-model climate simulations to analyze changes in the dependence structure between warm-season temperature and precipitation, showing that such dependence significantly increases the likelihood of concurrent hot and dry events [151]. An Earth System Model (ESM)-based study projected that under high-emission scenarios such as SSP5-8.5, CDHW events will persist for over 18 days with increasing intensity. Heatwaves are projected to be the dominant driver, and the coupling effects with drought are particularly pronounced in arid regions [157]. A study employed large ensemble climate model simulations to project future trends in precipitation and temperature over land regions, revealing that the occurrence of compound dry and hot months is primarily regulated by precipitation trends [162]. Most studies employing climate model simulation data to assess the occurrence probability and changing trends of CDHW events have yielded qualitatively consistent findings. Existing research highlights a projected increase in the frequency of CDHW events at both global and regional scales [11,155,158], growing exposure of populations and croplands [156], and a rise in the number of days that key crops are subjected to such extremes [103].

The simulation data generated by climate models require validation and calibration against meteorological observational data. After bias correction, climate model simulations generally agree well with observational results regarding the direction of changes in CDHW events. However, significant discrepancies remain in the magnitude of changes when comparing regional patterns and individual climate model outputs to observed variations in precipitation and temperature [10]. Moreover, some studies have highlighted that current climate models inadequately simulate the covariance between temperature and precipitation on short timescales [163]. To address these challenges, the use of multi-model ensembles from various GCMs is recommended to address modeling and scenario uncertainty. In addition, improving model resolution and physical process representation helps overcome limitations in model performance, enabling more reliable application of climate model data in CDHW-related studies.

### 5.2. Crop Model Simulation Data

Crop growth models are tools used to simulate crop growth and yield under various environmental conditions. These models account for factors such as weather, soil, and management practices to predict crop responses. They help researchers and farmers assess the impact of extreme weather events and evaluate adaptation strategies. In studying the effects of CDHW events on crops, some research utilizes crop model simulations. Compared to laboratory experimental data and field trial data, crop models allow researchers to efficiently simulate a range of scenarios, including CDHW events of varying timescales and intensities. They are particularly well-suited for modeling crop adaptation strategies under stress, such as adjusting sowing dates or selecting more drought- and heat-tolerant varieties. Additionally, when integrated with climate models, crop models can predict and assess the risks crops face from CDHW events under future climate conditions, providing valuable data on crop responses to these future scenarios. As previous studies have highlighted, a deeper understanding of the aboveground and belowground conditions plants experience in different scenarios makes modeling an effective tool for bridging the gap between controlled environments and field conditions, offering insights into how crops might adapt to extreme events [34].

Research on crop modeling to assess the impacts of CDHW stress on crops remains limited, but several significant studies have provided valuable insights. One study employed a parallelized DSSAT model to estimate maize, wheat, and soybean yields across the contiguous U.S. at a 5-arcminute resolution, showcasing DSSAT’s capacity for multi-crop and climate scenario simulations [150]. Another study used the GGCM EPIC-IIASA model, which replicates soil–plant–atmosphere biophysical processes, to simulate annual soybean yields within the ISIMIP phase 3a and GGCMI framework [8]. A comparison of crop yield models from 13 groups in the AgMIP-ISIMIP2a round demonstrated diverse modeling approaches to yield projections [144]. Additionally, the APSIM–Maize model, leveraging daily climate data, was applied to simulate maize responses to dry heat stress, affirming its adaptability to environmental stress modeling [27]. Similarly, a study in southeastern Australia used the APSIM model to simulate wheat responses to compound drought-heat events, highlighting the potential of adjusting sowing times and cultivar choices to mitigate the risks of such stresses under future climate scenarios [164].

## 6. Knowledge Gaps and Potential Future Works

### 6.1. Insights from Experimental Data at the Site Scale

Site-scale experimental data, including laboratory and field trials, provide direct information on crop responses to CDHW stress, influenced by stress intensity, vegetation type, habitat, and experiment settings [165]. Growth chamber experiments offer strict control over abiotic factors and yield efficient, reproducible data critical for understanding plant stress responses. However, a “glass wall” between laboratory and field researchers limits data integration and hinders the application of laboratory data on different genotypes and varieties under CDHW conditions to real agricultural systems [34,166].

Laboratory CDHW data mainly come from two approaches: one focusing on precision and reproducibility under fully controlled conditions, and the other controlling stress factors while simulating natural growth environments. Systematic comparisons between these datasets are lacking. Field trial data not only provide more direct insights for agricultural practices but also possess characteristics such as large-scale, multi-growing season, and multi-environment joint trials [75], which are often lacking in current lab data on CDHW stress. Few studies combine lab and field experiments; for example, a chili study screened for CDHW responses in growth chambers and then evaluated single-stress effects in the field [47], bridging controlled and field conditions. More such comparative studies are needed to improve understanding of CDHW impacts and support adaptation strategies.

A key limitation of current experimental data is that most studies apply only a single-stress treatment period and fixed stress levels, with few exploring the effects of initiating stress at different time points or incorporating varying degrees of stress severity [26,167]. This relatively limited experimental design may make it difficult to capture the full range of physiological and biochemical crop responses, failing to reflect the fluctuating and complex nature of real-world CDHW events, thus limiting the applicability of such data to agricultural systems. Future studies should incorporate a wider range of stress intensities across multiple growth stages to better simulate field conditions. Striking a balance between complexity and practical feasibility in experiments will help generate more comprehensive and reliable crop response data under compound stress.

The spatial propagation of CDHW extremes, along with the increased probability of such compound events [161] in a warming world, highlights the need for greater focus on obtaining crop response data under elevated CO_2_ conditions. Although a few studies have touched on this [60,70], much remains unexplored, particularly in the field trials. Using Free-Air CO_2_ Enrichment (FACE) systems enables researchers to increase CO_2_ levels in fields without disrupting the natural microclimate. Under these FACE conditions, researchers can investigate the effects of elevated CO_2_ on drought, heatwaves, and other variable conditions across the growing season [162]. Conducting CDHW compound stress trials within FACE environments holds significant promise for optimizing resources and facilitating the integration of large-scale, complex field trials in the future.

### 6.2. Insights from Big Data at the Large Scale

The extensive meteorological and Earth observation data provided by Earth observation systems, remote sensing systems, and climate monitoring systems [168], as well as the statistical and simulated data, mentioned in this review, all fall within the realm of big data. This review summarizes the specific types of big data and data collection channels involved in large-scale studies of CDHW events on crops.

Remote sensing big data has shifted the research field of climate extreme events away from reliance on traditional site-based measurements, enabling observations and estimates of key variables over larger spatial and temporal scales than ever before [169]. Compared to the widespread and comprehensive use of remote sensing data for monitoring and assessing individual drought and heatwave events, there remains a significant gap in its application to studying the impacts of CDHW (compound drought and heatwave) stress on crops. When monitoring and assessing CDHW events, the integration of multi-source remote sensing datasets is still insufficient. Building on the example set by various drought indices, developing new integrated monitoring indices for CDHW stress using multi-source remote sensing data, along with advancing more refined and targeted remote sensing-derived products to assess crop impacts, is a key area for future research.

Additionally, it is important to note that the application of crop model simulation data in studying the impacts of CDHW stress on crops remains insufficient compared to the more robust application of such data in single-stress simulations. Extensive research has been conducted using crop models to simulate the responses of crops to future climate change [170,171]. However, considerable efforts are still needed to effectively utilize crop models for studying CEs. In the field of studying CDHW stress on crops and their adaptations, only a limited number of studies have been conducted [164].

This research gap stems from the limited evaluation of different crop models’ abilities to simulate crop responses under compound drought and heat wave (CDHW) stress. While some models exhibit high sensitivity to moisture conditions critical for plant growth, others are more responsive to temperature fluctuations that impact crop development [172,173,174,175]. In the future, utilizing lab experiment data and field trial data to assess the capabilities of different crop models in simulating crop growth and development under CDHW stress presents a promising research perspective that can serve as the foundation for improving crop models and utilizing crop model simulation data under CDHW stress.

## 7. Conclusions

This review summarizes key data sources used to assess the impacts of CDHW stress on crops across different spatial and temporal scales. At the site level, experimental data reveal important crop responses but often lack coverage of diverse stress intensities and growth stages, and the gap between laboratory and field conditions hampers their integration.

At regional to global scales, meteorological observations, Earth system data, and statistical datasets have been widely and effectively used to assess CDHW impacts. However, the integration of remote sensing and meteorological data remains limited. Advancing multi-source data fusion and developing indices or products specifically tailored to CDHW conditions could significantly enhance the precision of large-scale crop impact assessments.

While crop models have been extensively used to evaluate the effects of individual stressors, their application under compound CDHW scenarios remains underdeveloped. More experimental data are needed to validate and improve model performance under these complex stress conditions. Enhancing crop models based on such data will be critical for accurately simulating compound disaster impacts in future research.

By clarifying the strengths and limitations of existing data sources and highlighting integration challenges, this review aims to support more effective data usage and promote cross-scale synthesis. It is expected to assist researchers in making informed choices about data application and encourage future efforts toward more integrated and accurate assessments of CDHW impacts on crop systems.
